# Contrasting Patterns of Genetic Structuring in Natural Populations of *Arabidopsis lyrata* Subsp. *petraea* across Different Regions in Northern Europe

**DOI:** 10.1371/journal.pone.0107479

**Published:** 2014-09-16

**Authors:** Mohsen Falahati-Anbaran, Sverre Lundemo, Stephen W. Ansell, Hans K. Stenøien

**Affiliations:** 1 Department of Biology, Norwegian University of Science and Technology (NTNU), Trondheim, Norway; 2 NTNU University Museum, Norwegian University of Science and Technology, Trondheim, Norway; 3 School of Biology and Center of Excellence in Phylogeny of Living Organisms, University of Tehran, Tehran, Iran; 4 Department of Ecology and Genetics, Evolutionary Biology Centre, Uppsala University, Uppsala, Sweden; 5 Department of Life Sciences, Natural History Museum, London, United Kingdom; North Carolina State University, United States of America

## Abstract

Level and partitioning of genetic diversity is expected to vary between contrasting habitats, reflecting differences in strength of ecological and evolutionary processes. Therefore, it is necessary to consider processes acting on different time scales when trying to explain diversity patterns in different parts of species' distributions. To explore how historical and contemporary factors jointly may influence patterns of genetic diversity and population differentiation, we compared genetic composition in the perennial herb *Arabidopsis lyrata* ssp. *petraea* from the northernmost parts of its distribution range on Iceland to that previously documented in Scandinavia. Leaf tissue and soil were sampled from ten Icelandic populations of *A. lyrata*. Seedlings were grown from soil samples, and tissue from above-ground and seed bank individuals were genotyped with 21 microsatellite markers. Seed bank density in Icelandic populations was low but not significantly different from that observed in Norwegian populations. While within-population genetic diversity was relatively high on Iceland (*H*_E_ = 0.35), among-population differentiation was low (*F*_ST_ = 0.10) compared to Norwegian and Swedish populations. Population differentiation was positively associated with geographical distance in both Iceland and Scandinavia, but the strength of this relationship varied between regions. Although topography and a larger distribution range may explain the higher differentiation between mountainous Norwegian relative to lowland populations in Sweden, these factors cannot explain the lower differentiation in Icelandic compared to Swedish populations. We propose that low genetic differentiation among Icelandic populations is not caused by differences in connectivity, but is rather due to large historical effective population sizes. Thus, rather than contemporary processes, historical factors such as survival of Icelandic lineages in northern refugia during the last glacial period may have contributed to the observed pattern.

## Introduction

Genetic diversity and structuring of natural populations are shaped by both historical and contemporary processes within a given region [1]. Much work has focused on the impact of local and regional habitat structure and landscape heterogeneity on levels of genetic diversity, differentiation, patterns of gene flow and adaptability of local populations [2]–[5]. Genetic connectivity between populations often varies across habitats [6] and different patterns of population differentiation may be expected among conspecific populations inhabiting regions in different parts of the distribution range [7]. Physical barriers and lack of biotic and abiotic dispersal vectors may reduce gene flow by preventing movement of either seed or pollen between populations [5]. Furthermore, limited gene flow over long relative to short distances creates an isolation by distance (IBD) pattern both under an island [8] and a stepping stone model [9], as well as increases adaptive differentiation between populations [10].

Population connectivity and genetic structure will, however, not only be shaped by physical features of landscapes and biological factors affecting the dispersal kernel [11]. Population history, including population age expressed as time since colonization, stability through time and historical effective sizes, may also influence observed levels of structuring [12], [13], and thus a diverse range of ecological and evolutionary processes must be examined in order to be able to explain patterns of biodiversity observed within species [14]. Pleistocene glaciations have affected the distribution and diversity of many taxa in central and northern Europe [15]. A number of studies have shown that populations experiencing rapid post-glacial colonization often exhibit different patterns of structuring compared to populations that survived the last glacial maximum *in situ* [16]–[18]. For instance, old populations may reach drift-gene flow equilibrium because of long time since colonization, resulting in a strong IBD pattern, whereas IBD is not always expected in recently established populations [7], [19]. Additionally, extent of geographical barriers, dispersal ability and proximity to refugia may influence the association of population age on strength and patterns of IBD [14], [20].

*Arabidopsis lyrata* is frequently used as a model for studies on genetic structuring, evolution of self-incompatibility and distribution in plants [21]–[26]. In addition, the disjunctive distribution of the species in central Europe, Scandinavia, Iceland and North America, with varying ecological and life history characteristics, have promoted studies of natural selection and the genetic basis of local adaptation [27]–[38]. Populations inhabiting sites with different ecological features and differing in life-history characteristics can also be useful in association studies to locate genomic regions associated with adaptive variation [34], [39]. Gaudeul et al. [40] recently demonstrated differences in genetic structuring and genetic diversity between contrasting mountainous and coastal populations of *A. lyrata* in Scandinavia, and hypothesized that variability in landscape structures may explain observed differences. In the Norwegian distribution range, the topography is rugged and dominated by valleys, whereas Swedish populations occur in a more open landscape along the coast. Mountains are expected to restrict dispersal of propagules in space, which can explain the higher between-population differentiation and lower genetic diversity in Norway compared to Sweden. Thus, if landscape structure has a significant impact on processes such as gene flow, a comparable pattern of genetic diversity and structuring might be expected for regions with similar landscape structures.

*A. lyrata* is distributed evenly throughout Iceland, and as opposed to Norway, there are fewer pronounced topographic barriers such as mountains, fjords and deep valleys to disrupt genetic connectivity among populations. The wide distribution of lava fields on Iceland, where *A. lyrata* act as a pioneer species [41], may help explain why the species is more common on Iceland than in most other regions in Europe, since the barren volcanic terrain will not necessarily act as a physical obstacle in restricting connectivity. Several studies of Icelandic *A. lyrata* using DNA sequence data, possibly subject to strong natural selection, show contrasting patterns of genetic differentiation on various geographical scales [21], [22], and overall gene flow levels on Iceland is difficult to infer from these studies. Both natural selection, gene flow, effective population size and age of populations will contribute to contemporary genetic structure, and non-neutral loci or neutral loci linked to selected sites may show contrasting patterns of genetic structure [42]. However, linkage between neutral and non-neutral loci in Icelandic populations has been found to be less likely due to the high frequency of recombination in a large region around the non-neutral loci in *A. lyrata* [43], [44].

It has been shown that life-history characteristics, such as presence of seed banks, may impact effective population sizes in Scandinavian populations of this [45] and related species [46]. It may therefore be important to take seed bank characteristics into consideration when trying to explain differences in variability patterns among different geographic regions. High levels of within-population variability and low levels of genetic differentiation in central European populations of *A. lyrata* has been attributed to glacial survival on the continent during the last glacial period [24]. Furthermore, demographic bottlenecks and the presence of a subset of genetic variants in populations from northern Europe and North America compared to populations from central Europe has been attributed to a post glacial colonization pattern [32]. On the other hand, relatively strong divergence between northern and central European populations has also suggested a cryptic Nordic refugium in this species [26].

In the present study we investigate whether patterns of genetic diversity within and among Icelandic populations of *A. lyrata* differ from that observed in Scandinavia. We want to quantify the relative impact of historical and contemporary processes in order to understand what factors explain the observed patterns of genetic diversity in northern Europe. In particular, we want to investigate to what extent observed patterns of genetic structuring are attributed to historical factors, like long-term survival and population stability through evolutionary time, and contemporary processes, involving dormancy, dispersal abilities and/or topographic features.

## Materials and Methods

### Ethics Statement

No specific permissions were required for the field activities on Iceland presented in this study. None of the study populations occurred within protected areas, and the study species itself is not endangered or protected. The GPS coordinates for the study populations are provided in Table 1.

**Table 1:** Description of localities for sampled Icelandic populations of *Arabidopsis lyrata*.

Population	Latitude	Longitude	Altitude	Ns	Na	Nsb	Habitat and soil type
Pop 2	65 09.001	021 03.561	34	92	31	2	Road side, Histic andosol
Pop 3	65 17.460	021 11.830	8	32	27	32	Road side, Andosol
Pop 4	65 23.835	021 11.737	21	29	23	2(1)	Road side, Histic andosol
Pop 5	65 19.661	020 39.776	112	32	27	6	Road side, Histic andosol
Pop 15	64 07.290	019 52.293	163	150	31		Road side, Sandy andosol with volcanic glasses
Pop 16	64 20.440	020 08.063	231	50	27		Road side, Brown andosols,
Pop 17	64 02.537	020 53.228	88	35	29	2	Road side, Leptosol/Sandy andosol complex
Pop 21	64 18.884	020 18.162	148	60	24	6(4)	Close to hot spring, Arable soil, Brown andosols,
Pop 22	64 50.440	021 19.521	80	40	19		River bank, Vitrisol
Pop 23	63 52.867	022 27.171	39	60	25	1(1)	Peat moss close to Blue Lagoon, Leptosol bed rock

Ns, Na and Nsb represent population census size, number of samples and number of seedlings from the seed bank, respectively. Numbers within parentheses indicate individuals that were not included in the microsatellite analysis. Altitude  =  m a.s.l.

### Study system

*Arabidopsis lyrata* ssp. *petraea* (L.) O'Kane and Al-Shebaz (Brassicaceae) is a perennial, self-incompatible herb with a highly disjunctive distribution in Europe, consisting of a narrow central European distribution area in Austria, Germany and the Czech republic, and a wide but fragmented northern European distribution area covering Iceland, Faeroe Islands, northern parts of United Kingdom, southwestern parts of Norway and the eastern coast of central Sweden [47], [48]. On Iceland, *A. lyrata* is widespread throughout the island (http://floraislands.is) and found in diverse habitats differing in several aspects from those in central Europe and the Scandinavian Peninsula. The island is dominated by few mountainous areas and extensive lowland lava plains, with the species growing under a range of conditions, from volcanic sands to rich soils in agricultural areas (Table 1). The species grows under a wide range of soil conditions on Iceland, varying from volcanic sands to rich soil of agricultural areas (Table 1).

Icelandic, Norwegian and Swedish localities vary in topography, vegetation cover, altitude and latitude. The Swedish populations are located at low altitude (<5 m a.s.l.) on the coast, covering a smaller geographical area (85×10 km; 62.583–63.183° N) [40], [47]. This in contrast to the large distribution area of *A. lyrata* on Iceland, where populations are located over a 300-km range [21]. In Norway, the species occurs from sea level up to 1,700 m a.s.l., and populations are separated by deep valleys and mountains over a large geographical area (385×180 km; 59.451–62.744° N). Differences in landscape topography among regions may lead to overall differences in wind speed among regions, and wind speed at local sites may also be strongly affected by microsite conditions. Lack of strong physical barriers and rather extensive lava fields may suggest wind to be as strong or stronger on Iceland compared to in the mountainous/forested regions of Norway and sandy beach and cliffy landscape of Sweden. To address this, average wind speed for different regions was acquired from meteorological stations nearby the sampling areas. Data for Norwegian, Swedish and Icelandic localities were obtained from the eKlima web portal of the Norwegian meteorological institute, http://www.tutiempo.net and http://en.vedur.is/climatology/data/, respectively. The average wind speed measured at meteorological stations in the study areas in Iceland, Sweden and Norway was 5.0, 3.0 and 2.8 m/s, respectively.

### Soil and leaf sampling

*A. lyrata* populations can form extensive seed banks [45], and as these may significantly increase contemporary and historical effective population sizes [45], [46], we were interested in investigating whether seed bank constituents differed between Iceland and that observed in Norwegian populations. We collected leaf tissue from the above-ground individuals from ten Icelandic populations within a 152×129 km area in the western parts of Iceland in August 2009 (63.881–65.397°N), at altitudes varying from 8 to 231 m a.s.l. (Table 1). Most of the populations included here (n = 6) have previously been studied by Schierup et al. [21] to investigate the geographical distribution of nucleotide variation at the self-incompatibility (SI) locus. Due to the widespread distribution pattern and the relatively open topography of Icelandic sites, it seems plausible that local sites included in this analysis form metapopulation systems; a study by Lundemo et al. [49] showed that small levels of genetic differentiation can arise among patches within a local population of *A. lyrata*.

To examine whether Icelandic populations form a soil seed bank, ten soil samples of 10 cm×10 cm, with a depth of 5 cm, were taken throughout each of the studied populations before the annual seed rain. However, only five samples could be collected from the spatially very restricted population 22. Previous germination trials of field collected seeds from Norway have shown that no stratification is required for successful germination (S. Lundemo, unpublished results). However, to ensure germination of as many seeds as possible, soil samples were stored at 4°C for at least one month. Soil samples were spread out in a thin layer (1 cm) on top of commercial potting soil in the greenhouse and germinated at 16 h day light, 20°C and 65% air humidity. Seedling emergence from the soil was monitored over a period of ten months, and all seedlings germinated within two months after sowing. At each monthly census, new seedlings were counted and removed. Leaf tissue from each seedling was collected and dried at 45°C overnight. Seed bank density was determined by dividing the total number of seedlings emerging from the soil by total volume of soil for each population, following Falahati-Anbaran et al. [45].

### Microsatellite analysis

Genomic DNA was extracted from leaf tissue using E-Z 96 Plant DNA Kit (Omega Bio-Tek, GA, USA). Twenty-one primer pairs labeled with different fluorescent dyes were used in 3 separate multiplex PCR reactions (Table S1 in File S1). The total volume of each PCR reaction was 10 µl; containing 5 µl 2x Type-it ™ Microsatellite PCR Kit (Qiagen, Hilden, Germany), 1 µl primer mix (10x) and 1 µl template DNA. A more detailed description of the PCR conditions and fragment analysis protocol can be found in [45].

### Statistical analyses

Soil seed banks contribute to preservation of genetic variation in natural populations of *A. lyrata* [45], and it is therefore of interest to determine the extent and magnitude of seed banks in different regions when comparing patterns of genetic structuring. A Mann-Whitney U test was used to compare the seed bank density in Icelandic populations to that previously reported for 14 Norwegian populations [45]. The number of seeds in the soil may be a function of the above-ground plant density and to examine this, a non-parametric correlation test (Spearman's *ρ*) was used to evaluate the association between soil and above-ground density. In total, 308 samples were obtained from above-ground and seed-bank cohorts and genotyped using 21 microsatellite markers. Because we did not find significant differences in seed bank density between Iceland and Norway (see below), further analysis of Icelandic samples was limited to the 263 above-ground individuals. Identical multilocus genotypes within populations were removed, i.e., only one individual from each multilocus genotype (genet) was included in the genetic analyses per population in order to avoid estimation bias due to clonality [40]. Following this procedure, 15 individuals were found to be likely the result of clonal propagation, and therefore excluded from the data set, leaving 248 individuals for further analyses. The probability that two individuals with identical multilocus genotypes would be present in a population (PI) was calculated as 2(∑P_i_^2^)^2^ -∑P_i_^4^ [50], where p_i_ is the frequency of the i^th^ allele at a given locus and multiplied over all loci. The calculated PI in the study population was less than 1.6×10^−8^.

The 248 individuals were subsequently used to compare patterns of genetic variability with the Scandinavian populations included by Gaudeul et al. [40], who also only used above-ground material for genetic analyses. The proportion of genets (G/N) was calculated for each population by dividing the number of unique individuals (G) by total number of genotyped samples (N). For each population, the average observed (*H*_O_) and expected heterozygosity (*H*_E_) over all loci was estimated using Genepop version 4.1 [51]. To test for Hardy-Weinberg equilibrium, the global exact test for heterozygote deficiency was carried out by estimating the exact *P*-value using a Markov Chain algorithm [52]. *P*-value for multiple comparisons was corrected with the sequential Bonferroni correction [53]. Because the presence of null alleles may lead to an increase in the homozygote frequencies compared to that would be expected under Hardy-Weinberg equilibrium, the frequency of null alleles was computed using an expectation maximization algorithm [54] implemented in the program FreeNA [55]. Inbreeding coefficient (population specific *F*_IS_) and the statistical significance of *F*_IS_ was determined by 10,000 random permutations of alleles between individuals using Arlequin 3.5 [56]. The proportion of polymorphic loci (PPL) was calculated by dividing the number of polymorphic loci by the total number of loci for each population. Allelic richness (*R*_S_), the number of alleles adjusted for variation in sample size, and private allelic richness were estimated over all loci for each Icelandic population using a rarefaction method implemented in HP-RARE 1.0 [57]. The squared differences in lengths between two alleles at a locus averaged over all loci (*md* ^2^) was calculated for each individual [58] and average *md* ^2^ across individuals was reported for each population. Neutral alleles at a locus will become progressively more differentiated from one another over evolutionary time due to stepwise mutations in SSR (simple sequence repeat) markers, and a positive relationship between *md^2^* and time since divergence is expected [58]. We also estimated *H*_E_, *F*_IS_, *R*_S_, *R_P_* and *md^2^* for Norwegian and Swedish populations based on previously published data [40] using 17 loci which were common with the Icelandic dataset (Table S1 in File S1). Analysis of variance was used to examine regional differences for *H*_E_, *F*_IS_, *R*_S_, *R_P_* and *md^2^*. Multiple comparisons between regions were performed using a post hoc Bonferroni test. All parameters were tested for normality and homogeneity of variance prior to analysis, and *md^2^* was square root transformed prior to analysis in order to meet assumptions of the test. The average frequency of null alleles across populations for all loci and between regions was compared using a paired *t*-test.

To detect recent declines in effective population sizes due to bottlenecks, a one-tailed Wilcoxon signed rank (WSR) test with sequential Bonferroni correction of *P*-values was performed to assess genetic diversity excess under mutation–drift equilibrium relative to that expected from the number of alleles in a sample. The two-phase mutation model (TPM) was used for probability estimation based on 10,000 replications as implemented in Bottleneck version 1.2 [59]. In the TPM model, the proportion of stepwise and multi-step mutations for TPM was set to 70% and 30%, respectively. The program computes the expected equilibrium heterozygosity (*H*eq) from the number of alleles for each locus and population, and tests the deviation of *H*eq from observed gene diversity (*H*_E_). The bottlenecked populations should show a decline in both the number of alleles and *H*eq under mutation drift equilibrium, and thus *H*eq might be smaller than *H*_E_ [60].

Genetic structure of Icelandic populations was assessed by computing pairwise *F*_ST_ among populations. The magnitude of regional *F*_ST_ can reflect the strength of the homogenizing effect of gene flow among populations in each region. To investigate regional differences in population differentiation, pairwise *F*_ST_ between Icelandic populations was compared to that in Scandinavian populations. Due to non-independence of among-population *F*_ST_ values, we applied a randomization test following a one-way analysis of variance (ANOVA) to test the null hypothesis of no difference between regions using EcoSim [61]. The presence of null alleles may bias estimates of population differentiation, and unbiased *F*_ST_ measures accounting for null alleles were computed using FreeNA [55]. These measures were then compared to the uncorrected *F*_ST_ for each region and one-way ANOVA was used to test for differences. For each comparison, a pseudo *F* ratio similar to a standard *F* ratio was computed and compared to the simulated *F* ratio calculated using a randomization test with 1,000 iterations to determine the probability that the observed *F* ratio was greater or less than that expected by chance. To test whether variation in size of distribution area inflates genetic differentiation levels, we limited the analysis to populations from areas of similar sizes and population numbers across regions. For this, five populations from each area were selected; Iceland (15, 16, 17, 22 and 23), Sweden (S3, S6, S10, S15 and S19) and Norway (N5, N6, N8, N9 and N10). In order to test for isolation by distance, the relationship between log_10_ [*F*_ST_/(1-*F*_ST_)] and geographical (log_10_ transformed) distances was studied using a Mantel test implemented in GenAlEx 6.4 [62]. To test whether the pattern of isolation by distance differed among regions, i.e., differing slopes of regression lines (b), an analysis of covariance (ANCOVA) was conducted.

Genetic structure of Icelandic populations was also investigated using a Bayesian clustering analysis as implemented in Structure 2.3.3 [63], and the computations were conducted in https://lifeportal.uio.no/. A model of admixture together with correlated allele frequencies was applied for parameter estimation using 2×10^5^ Markov Chain Monte Carlo (MCMC) iterations, following a burn-in period of 1×10^5^ iterations. The likely number of clusters was obtained by plotting the average ln probability of data [ln P(D)] over 10 independent runs for *K* = [1]–[10]. We also calculated Δ*K* based on an ad hoc method to determine the conservative minimum number of clusters [64]. The matrices of membership coefficients across 10 independent runs were used to search for the optimal alignment using a greedy algorithm with 1×10^5^ permutations in CLUMPP version 1.1.2 [65]. Membership coefficients of individuals to the inferred clusters was plotted for each *K* using DISTRUCT version 1.1 [66].

To investigate the importance of evolutionary processes on patterns of genetic structuring, we estimated immigration rates and effective population sizes (*N*_e_) for each population. The rate of contemporary immigration into populations was estimated as the probability that individuals belong to their respective populations, and computed with a Monte-Carlo resampling of 1×10^4^ individuals using a Bayesian approach implemented in GeneClass2 [67]. Individuals with assignment probability >0.01 to its respective population were considered to be residents, otherwise individuals were considered immigrants [68]. The mutation-scaled historical effective population size (*θ*) and immigration rate (*M*) were simultaneously estimated using a coalescence-based maximum likelihood approach implemented in Migrate 3.2 [69], [70]. The mutation-scaled migration rate (*M* = *m/u*, where *m* and *u* are immigration rate and mutation rate per generation, respectively), reflects the average rate of immigration relative to mutation rate through historical time. Migrate assumes constant population size over time, a symmetric migration rate between populations and that all populations exchanging genes have been sampled [70]. Unsampled populations should have a negligible impact on *M* = *m/u*, but can bias the effective population size estimates upward [71]. A Brownian motion approximation to the stepwise mutation model was applied and MCMC simulation was conducted with 10 short chains (sampling at 1×10^4^ trees) following one long chain (sampling at 5×10^5^ trees). The simulation was repeated five times to improve the accuracy of results as suggested by Beerli and Felsenstein [69] and the average over five replicates with similar parameter setting was reported for *θ* and *M*. In addition, *θ* and *M* were estimated based on the same markers for Scandinavian populations. The historical *N*_e_ demonstrates the long term effect of evolutionary processes such as mutations and random genetic changes on genetic variation. Under mutation-drift equilibrium the amount of genetic variation is expected to be *θ* = 4 *N*_e_ *u* in a diploid population, where *θ*, is four times the product of the effective population size (*N*_e_) and mutation rate per generation (*u*) for microsatellite loci. Historical *N*_e_ can be estimated by calculating *θ* and dividing by estimates of *u*, or compared between regions by dividing *θ* estimates, thereby canceling out the mutation rate, i.e., assuming that neutral mutation rates do not vary across the distribution range. Analysis of variance was conducted to compare *θ* and *M* between regions, and multiple post hoc comparisons based on the Bonferroni method was used to test for differences between regions. All statistical tests were conducted using SPSS version 16.0.

## Results

The seed banks of the Icelandic populations contained on average (± SE) 26.8±16.6 seedlings/m^2^ soil sampled (median 7.59, CV = 196%, Table 2). Although this was considerably smaller than in Norwegian populations (average  = 67.3 seedlings/m^2^, median  = 23.60, C.V. = 136%; [45], a Mann-Whitney U test revealed no significant difference between regions (*U* = 41; n_total_ = 24, two-tailed *P* = 0.088). The percentage of populations either lacking a seed bank entirely or with <10 seedlings/m^2^, were 21% and 70% for Norway and Iceland, respectively (Table S2 in File S1). The above-ground density of plants on Iceland (average number of individuals/m^2^ ± SE) was 1.38±0.58, (median  = 0.64, C.V. = 133), not significantly different from that observed in Norwegian populations (average ± SE  = 3.09±1.19, median  = 1.17, C.V. = 125, *U* = 40, two-tailed *P* = 0.186, Table S2 in File S1). There was no significant relationship between density of above-ground plants and seed bank density in Icelandic (Spearman's *ρ* = 0.46, *P* = 0.18) or Norwegian (Spearman's *ρ* = 0.36, *P* = 0.20) populations.

**Table 2:** Summary of genetic diversity parameters across seventeen microsatellite loci, above-ground plant (m^−2^) and seed bank density (seedlings/m^2^) in Icelandic populations of *Arabidopsis lyrata*.

Population	*H*_O_	*H*_E_	*F*_IS_ a	PPL	*R*_S_	Number of plants/m^2^	Seedlings/m^2^
Pop 2	0.35	0.35	−0.01	0.76	2.28	0.12	9.8
Pop 3	0.42	0.34	−0.27	0.71	2.03	5.11	172.4
Pop 4	0.41	0.39	−0.05	0.88	2.48	0.19	5.4
Pop 5	0.40	0.36	−0.17	0.71	2.55	4.51	32.3
Pop 15	0.38	0.36	−0.07	0.82	2.41	0.43	0
Pop 16	0.34	0.34	−0.03	0.76	2.22	0.67	0
Pop 17	0.38	0.34	−0.13	0.76	2.11	0.23	10.8
Pop 21	0.40	0.37	−0.08	0.71	2.23	1.20	32.3
Pop 22	0.31	0.33	0.05	0.76	2.17	0.61	0
Pop 23	0.31	0.32	0.02	0.76	2.31	0.74	5.4
Average	0.37	0.35	−0.08	0.76	2.28	1.38	26.8
SE	0.01	0.01	0.03	0.02	0.05	0.58	16.6

a None of the *F*_IS_ values were significantly different from zero, indicating random mating within populations (*P*>0.05).

*H*_O_, *H*_E_, *F*_IS_, PPL and *R*_S_ represent observed heterozygosity, genetic diversity, inbreeding coefficient, proportion of polymorphic loci and allelic richness, respectively.

### Differences in within-population genetic diversity between regions

In total, 134 alleles were detected across 21 microsatellite loci in Icelandic populations, with the average (± SD) number of alleles per locus being 6.38±5.92 (range 1–26). Four loci (*ELF*3, *ICE*3, *MSAT*2.22 and *nga*112; Table S1 in File S1) were excluded from estimation of genetic diversity parameters in order to enable comparisons with previous estimates from Norway and Sweden [40]. Because of the lack of significant differences in seed bank parameters between Iceland and Norway, we excluded genotypic data from seed banks from subsequent genetic analyses, enabling a direct comparison with results presented in [40]. In total 77 alleles were scored in the 17 loci from above-ground Icelandic plants, and the average number of alleles was found to be 4.53±3.34 (range 1–14, Table S1 in File S1). The average proportion of genets (± SE), G/N was 0.94±0.02. All loci except *nga*162 were polymorphic in at least one population, and the average proportion of polymorphic loci (PPL) over all Icelandic populations was 0.76±0.02 (± SE). Estimates of *H*_O_, *H*_E_, *F*_IS_, PPL, *R*_S_ and *R*_P_ for each population are presented in Table 2. Genetic diversity (*H*_E_) varied considerably among loci (range 0.008–0.643), but less between Icelandic populations, with an average of 0.35 (range 0.32–0.39), with no populations showed significant heterozygote deficiency after Bonferroni corrections (*P*>0.005). None of the *F*_IS_ values were significantly different from zero (*P*>0.05, Table 2). Three loci (*ICE6*, *ICE7* and *F19K23-483*) showed heterozygote deficiency after sequential Bonferroni correction (*P*<0.0035), probably due to presence of null alleles. Additionally, the average (± SE) frequency of null alleles across populations over all loci was 0.02±0.004, and only three loci (*ICE6*, *ICE7* and *F19K23-483*) exhibited a null allele frequency higher than 0.05. Average (± SE) allelic richness and private allelic richness over all populations was 2.28±0.05 (range 2.03–2.55) and 0.13±0.03 (range 0.01–0.26), respectively. The mean *d* (*md* ^2^) (± SE) over all Icelandic populations was 32±1.7. Bottleneck tests revealed a signature of recent reduction in effective size in three populations (3, 17 and 21) after sequential Bonferroni correction on the *P*-value, with significant heterozygosity excess compared to expectations under mutation-drift equilibrium based on the two-phased model (TPM).

Analysis of variance revealed a significant difference among regions for genetic variation (*H*_E_; *F*_2,33_ = 20.46, *P*<0.001), allelic richness (*R*_S_; *F*_2,33_ = 4.7, *P* = 0.016), private allelic richness (*R*_P_; *F*_2,33_ = 5.06, *P* = 0.012), and *md* ^2^ (*F*_2,33_ = 32.65, *P*<0.001), but no difference in *F*_IS_ (*F*_2,33_ = 0.10, *P* = 0.41; Table S2 and Figure S1 in File S1). The mean (± SE) standing genetic variation (*H*_E_) in Icelandic (n = 10, 0.35±0.007) was similar to Swedish (n = 12, 0.33±0.015) populations and both were significantly higher than Norwegian populations based on post hoc Bonferroni test (n = 14, 0.25±0.011; *P*<0.01). Pairwise comparison revealed that allelic richness, *R*_S_, was significantly higher in Icelandic populations (average ± SE, 2.28±0.05) than in Norwegian (average ± SE, 2.03±0.07; *P* = 0.027) populations, and similar to Swedish populations (average ± SE, 2.23±0.06). Private allelic richness was significantly higher in Icelandic (average 0.13±0.030) compared to Norwegian (average 0.05±0.01; *P* = 0.032) and Swedish (average 0.05±0.02; *P* = 0.020) populations, whereas Norwegian and Swedish populations did not differ from each other (*P*>0.1). *Md* ^2^ in Icelandic populations was significantly higher than that in Norwegian (average 13.58±1.66; *P*<0.01) and Swedish (average 19.42±1.53; *P*<0.01) populations based on post hoc Bonferroni tests. The average (± SE) frequency of null alleles for all loci across Icelandic populations (0.02±0.004) was similar to that for Norwegian (0.02±0.005, *t* = 1.35, two-tailed *P* = 0.19) and Swedish populations (0.03±0.004, *t* = 1.02, two-tailed *P* = 0.32). No significant difference was observed between Norway and Sweden (*t* = 0.07, two-tailed *P* = 0.95)

### Spatial genetic structure across regions

**Figure 1:**
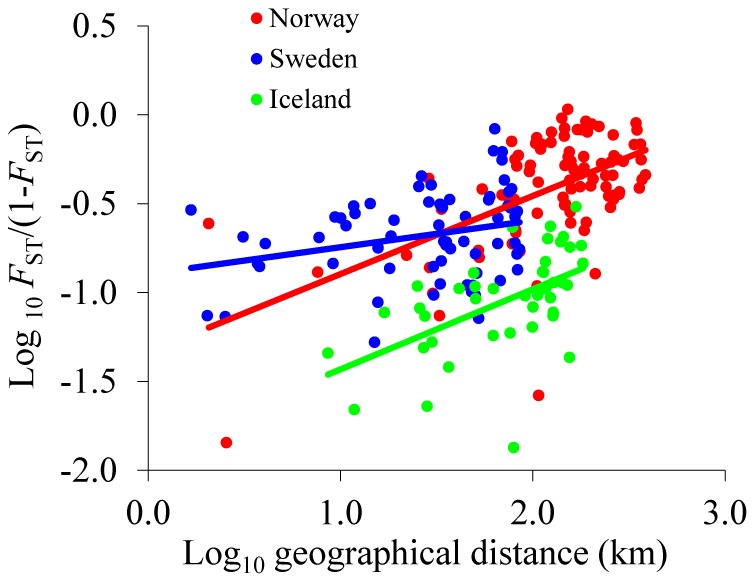
Relationship between genetic differentiation (log_10_ [*F*_ST_/(1-*F*_ST_)]) and geographic distance (log_10_ transformed) in *Arabidopsis lyrata*. Blue, red and green represent Swedish (r = 0.27, n = 66, *P* = 0.008), Norwegian (r = 0.57, n = 91, *P* = 0.001) and Icelandic ( = 0.55, n = 45, *P* = 0.001) populations, respectively.

The average *F*_ST_ (± SE) among Icelandic populations was 0.10±0.01 (range 0.010–0.23). One-way ANOVA revealed a significant difference among regions in levels of genetic differentiation. The observed *F* ratio for Norway-Sweden, Norway-Iceland and Sweden-Iceland comparisons were 46.60, 133.80, and 44.28, respectively (all *P*<0.001). The unbiased *F*_ST_ corrected for null alleles was similar to uncorrected *F*_ST_ for Icelandic (observed *F* ratio  = 0.03, *P* = 0.87), Norwegian (observed *F* ratio  = 0.13, *P* = 0.71) and Swedish (observed *F* ratio  = 0.05, *P* = 0.83) populations. The average *F*_ST_ (± SE) was higher in Norwegian (0.30±0.12, range 0.01–0.52) than Swedish populations (0.19±0.01, range 0.05–0.45, *P*<0.001), which in turn was higher than in Icelandic populations (*P*<0.001). Geographical and genetic distances were significantly correlated in the samples from Iceland (r = 0.55, n = 45, *P* = 0.001), Norway (r = 0.57, n = 91, *P*<0.001), and Sweden (r = 0.27, n = 66, *P* = 0.008; Figure 1). Analysis of covariance revealed a significant country × geographical distance interaction, indicating dissimilar regression slopes between countries (*F*_2,196_ = 5.37, *P* = 0.005). The regression slope of Swedish populations (b = 0.15) differed significantly from Norwegian (b = 0.44; *F*_1,153_ = 9.00, *P* = 0.003) and Icelandic (b = 0.45; *F*_2,107_ = 5.70, *P* = 0.019) populations, whereas the two latter regions did not differ (*F*_1,132_ = 0.007, *P* = 0.92). We also investigated genetic differentiation by including five populations at similar geographic scales in Icelandic (mean between-population distance  = 56.0 km, range 8.6–106), Norwegian (mean  = 60.7 km, range 2.6–82.6), and Swedish populations (mean  = 46.3 km, range 9.1–85.3). This approach resulted in average *F*_ST_ estimates being reduced to 0.07, 0.17 and 0.20 for Icelandic, Swedish and Norwegian populations, respectively, but regions were still significantly differentiated (*F*_1,27_ = 7.23, *P*<0.001). Accordingly, a post hoc Bonferroni test revealed that the population differentiation in Iceland was significantly lower than that in Norway (*P* = 0.003) and Sweden (*P* = 0.019), but the latter regions did not differ significantly from each other (*P* = 0.932).

**Figure 2:**
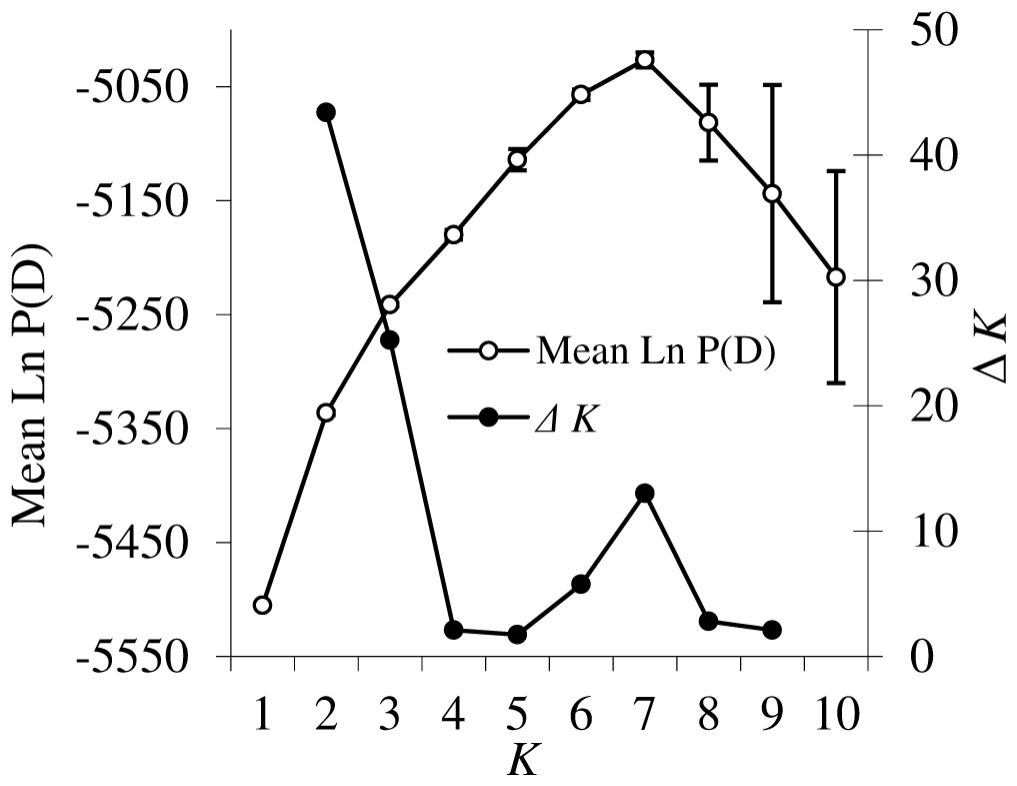
Mean probability of data (open circle) and Δ*K* (closed circle) obtained by Structure in ten Icelandic populations of *Arabidopsis lyrata*. Vertical lines indicate the standard deviation for the mean probability of data.

**Figure 3:**
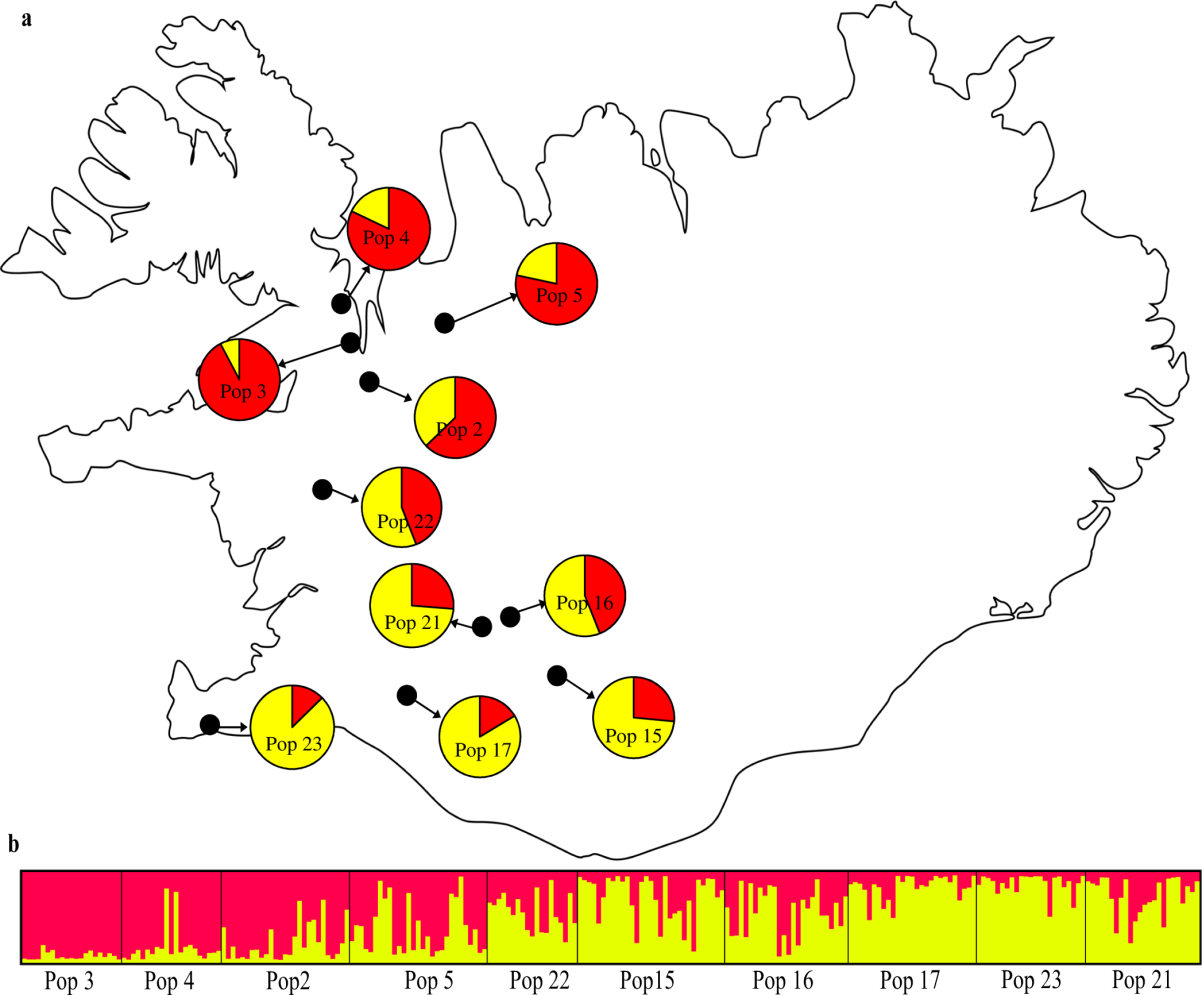
Genetic structure of Icelandic populations of *Arabidopsis lyrata* detected by Structure. a) Each pie represents membership coefficients of each population to the inferred cluster (*K* = 2). b) Membership coefficients of individuals to the inferred clusters. Solid vertical lines separate populations.

The optimal number of clusters in Icelandic populations using Bayesian clustering revealed a peak for Δ*K* at *K* = 2 based on the method [64] (Figure 2), splitting the northern and southern populations (Figure 3a, b). However, based on the mean ln P(D), the highest peak, representing the optimal number of clusters, was observed for *K* = 7 (Figure 2). The results based on *K = 7* showed that populations are geographically structured into several ancestral genetic clusters (Figure S2a, b in File S1).

### Immigration rate and historical effective population size

Bayesian analyses showed that none of the populations from the different regions had experienced a recent immigration event (Figure S3 in File S1). The average historical immigration rate (mutation-scaled migration rate; *M* ± SE) was 1.06±0.03, 1.05±0.05 and 0.95±0.03 per generation for populations from Iceland, Sweden and Norway, respectively, implying that migration and mutation rates are similar. Furthermore, no significant difference was found among the regions for historical migration rates (*F*_2,33_ = 2.56, *P* = 0.093). The average (± SD) historical effective population size (estimated as *θ*) was 0.46±0.03 in Icelandic populations, 0.29±0.05 in Norway and 0.32±0.07 in Sweden (Table S2 in File S1). Historical *N*_e_ differed significantly between regions (*F*_2,33_ = 19.42, *P*<0.01, Figure S1 in File S1), and a Bonferroni test indicated significantly higher historical *N*_e_ in Icelandic than Norwegian and Swedish populations (*P*<0.01).

## Discussion

### Variation in ecological characteristics

Old and stable populations may contain more allelic variants than short-lived, unstable populations. Furthermore, ecological features like soil seed banks may contribute to maintaining genetic diversity by preserving alleles descended from multiple generations in the past, resulting in increased effective sizes of plant populations [45], [46], [72]. Despite a similar sampling regime, the percentage of populations lacking a seed bank in Iceland (30%) is seemingly twice as high compared to a previous study of Norwegian populations (14%) [45]. Our results thus indicate that Icelandic populations maintain small seed banks relative to the Norwegian populations of *A. lyrata,* although the difference is not statistically significant. Seed bank densities observed in our study are similar to those documented for *A. lyrata* by Marteinsdóttir et al. [73] in an outwash plain in southern Iceland (average  = 27 seeds/m^2^). Moreover, above-ground plant density do not differ between the countries [45]. In our study, it seems that above-ground plant density does not affect density of ungerminated seeds in soil seed banks, and other factors such as soil substrate, dormancy, percentage of adult plants and/or reproductive output can be more important in this respect (cf. [73], [74]). Since our results show no significant differences between Iceland and Scandinavia regarding seed bank characteristics, we have compared the above-ground statistics for various regions assuming that the differences observed for genetic parameters are likely not due to differences in seed dormancy patterns.

### Within-population genetic diversity and spatial genetic structure

The average genetic diversity in Icelandic populations (*H*_E_ = 0.35) is similar to what is found in Swedish populations (*H*_E_ = 0.33; [40], which in turn is higher than that observed in Norwegian populations (*H*_E_ = 0.25). This is consistent with Muller et al. [23], who also found a higher level of genetic diversity in Icelandic than Norwegian populations, based on results from one and two populations from Iceland and Norway, respectively. Most of the genetic variation in Icelandic *A. lyrata* is partitioned within populations, with a low but significant level of among-population differentiation (average *F*_ST_ = 0.10). The level of genetic differentiation among Icelandic populations (mean distance  = 93 km) is ∼2- and 3-fold lower than that in Swedish (mean distance  = 40 km) and Norwegian (mean distance  = 165 km) populations, respectively. Furthermore, the level of differentiation in Icelandic populations remains significantly lower than Norwegian and Swedish populations after adjusting for geographical scale and population number across regions. This suggests that the wider distribution area on Iceland cannot explain differences in population differentiation between the studied regions.

Our sampling regime has covered only the western parts of Iceland, but this has probably not had much or any impact on our estimates of genetic structure in Icelandic populations. It is worth noting that the Icelandic populations used for between region comparisons accounting for similar sized distribution area (see material and methods), exhibit similar levels of genetic diversity within populations compared to all Icelandic populations sampled in our study. Thus, the range of within-population diversity is narrow (*H*_E_ = 0.32–0.39) on Iceland compared to Norwegian (*H*_E_ = 0.–0.34) and Swedish (*H*_E_ = 0.21–0.38) populations. This suggests that increasing the sampling efforts to more than ten studied populations will likely not change our estimates.

Homogeneous and continuous habitats may facilitate gene flow and reduce genetic differentiation between populations [19], though it is not straightforward to assess to what degree differences in dispersal vector efficiency vary among studied regions. The sparse tree cover and lack of strong topographic barriers over large geographical areas on Iceland can potentially cause wind to act as a dispersal agent and facilitate seed dispersal over longer distances compared to that in more closed vegetation types. However, several factors make it difficult to conclude on this. First, even though wind speed measured at meteorological stations on average is stronger on Iceland compared to in Swedish and Norwegian study areas, it is not given that efficiency of wind at canopy level is any different among regions. Microsite wind patterns can be different from average measures on local or regional levels, and e.g. localities in Norwegian mountain are expected to be strongly influenced by wind. Second, and perhaps more importantly, as *A. lyrata* is insect pollinated [41], [75], high wind speed could influence pollen flow negatively by restricting pollinator activity [76]–[78].

The wider distribution area and rugged mountainous landscape in the Norwegian compared to the Swedish distribution area could explain why population differentiation is higher among Norwegian relative to Swedish populations [40]. The increased population differentiation as a function of geographical distance suggests that dispersal via pollen or seed is distance dependent, i.e., with a higher level of gene flow at shorter relative to longer distances [7]. The IBD pattern for different regions shows that increased differentiation as a function of distance is stronger in Norwegian and Icelandic relative to Swedish populations (Figure 1). These results indicate that pollen and/or seed dispersal is more distance-restricted in Icelandic and Norwegian populations relative to Swedish populations [41]. *A. lyrata* seeds are primarily gravity-dispersed without specialized mechanisms for long-distance dispersal in open habitats, and large distribution areas on Iceland and perhaps Norway during or after the last glacial period may explain the difference in IBD patterns to that observed in Swedish populations. Additionally, multiple colonization routes into the Norwegian and Icelandic distribution ranges may account for the observed differences in the IBD pattern [79], [80].

It should be noted that homoplasy and presence of null alleles in microsatellite loci may influence population genetic estimates [81]. Allele size homoplasy has been reported for some microsatellite loci in *A. lyrata,* and Muller et al. [23] suggest that size homoplasy may bias estimates of between region divergence. We find frequency of null alleles to be consistently low in populations from all three regions, and these alleles have no detectable effect on estimates of population differentiation. It has been argued that the presence of size homoplasy will be problematic while reconstructing phylogenetic history, but has been found to have no significant effect on population parameters because the effect is compensated by high variability at microsatellite loci [82].

### Historical and contemporary processes in different regions

We observe that the historical effective population size (*N*_e_) in the Icelandic region is considerably larger than in Swedish populations while levels of genetic diversity are similar. On the other hand, Icelandic populations exhibit higher allelic richness and genetic diversity, and has larger effective population size compared to the Norwegian region. The larger effective population size of Icelandic than Swedish populations may be related to differences in private allelic richness between regions, possibly explained by older age of the Icelandic compared to the Swedish metapopulation. Interestingly, we find that the average mutation-scaled migration rate (*M*) do not differ between regions, suggesting similar rates of immigration over time in populations of different regions. In addition, we find no evidence of contemporary migration into populations in different regions using an assignment test, suggesting limited gene flow on both ecological and evolutionary time-scales.

Mean d^2^ (*md^2^*) has been used as measure of divergence time between lineages within populations over time [58]. A higher *md^2^* in Icelandic populations compared to Norwegian and Swedish populations suggests longer coalescence times for Icelandic allelic variants, and hence possibly older populations on Iceland than in Scandinavia. Even though there is a significant difference between regions for *md^2^*, the presence of null alleles may negatively bias *md^2^* by increasing the observed frequency of homozygotes. However, since the frequency of null alleles is low and similar between regions, this may not be influencing the patterns observed in the present study. Additionally, *md^2^* is sensitive to inbreeding patterns [83], but this is also likely not an issue here since all populations exhibit *F*_IS_ equal to zero. Finally, the extent of allele length differences could be due to admixture of divergent lineages, for instance central European and Icelandic lineages in relatively recent time (cf. [26]), but several observations argue against this possibility. First, recent admixture should be reflected in subsequent heterozygote deficiency over studied loci, and despite indications of heterozygote deficiency at three loci when analyzing all samples together (probably due to null alleles), we find no evidence of heterozygote deficiency at the population level. Second, immigrant individuals carrying other genotypes than in residents might be expected to have a low probability of assignment to the population to which it has immigrated [68]. Our assignment analysis shows no overall differences in the pattern of assignment probability between regions, and seemingly no indication of differences in recent admixture patterns. Thus, contrasting patterns of population differentiation between regions seems more likely due to glacial survival of Icelandic populations and differences in the demographic histories of populations, including larger historical *N*_e_ in Icelandic than Norwegian and Swedish populations, than migration rates *per se* [26]. Several studies using genetic and paleobotanical data have suggested glacial survival of plant species in Icelandic and Scandinavian refugia [84]–[86]. Interestingly, the presence of seeds of *A. lyrata* ssp. *petraea* has also been recorded in the Ballybetagh bogs near Dublin in Ireland and dated to the end of last glacial period [87], and a recent study using nuclear genes have suggested that northern European populations of *A. lyrata* diverged before the last glacial maximum [88].

## Conclusions

Our results show that there is large variation in genetic diversity, population structure and effective sizes in natural populations of *A. lyrata* inhabiting three topographically contrasting regions in its northernmost European range. Population differentiation in Icelandic populations is unexpectedly low, even though immigration rates do not differ between regions. The observed low population differentiation can be due to large effective population size rather than differences in historical or ongoing gene flow between populations. Interestingly, Ansell et al. [26] and Vigueira et al. [88] found indications that the species may have survived last glacial maximum in a cryptic northern refugium. One may thus hypothesize whether the higher effective size of Icelandic populations reflects old ages of lineages residing in stable refugial populations. The strong isolation by distance pattern observed in our study is in contrast to results reported by Schierup et al. [21], suggesting a recent colonization of Icelandic populations because of absence of IBD and low population differentiation at the self-incompatibility locus. However, it seems possible that this could be due to frequency-dependent selection and rapid homogenization of rare S-alleles [21], as opposed to likely neutral or near-neutral evolution at microsatellite markers. To conclude, we find that Icelandic populations express different patterns of genetic structuring compared with Scandinavian populations, probably representing an older colonization history than that previously assumed and possibly reflecting relatively more stable population sizes through time.

## Supporting Information

Supporting Information S1 **File contains Tables S1 and S2 and Figures S1, S2, and S3.** Table S1. Characteristics of microsatellite loci used to describe the genetic structure in natural populations of *Arabidopsis lyrata* in Iceland. Table S2. Above-ground plant and seed bank density, and estimates of genetic parameters based on 17 microsatellite markers in Icelandic, Norwegian and Swedish populations of *Arabidopsis lyrata*. Figure S1. Heterozygosity (*H*_E_), effective population size (*N_e_*), allelic richness (*R*_S_) and private allelic richness (*R*_P_) in Icelandic, Norwegian and Swedish populations. Figure S2. Genetic structure of Icelandic populations of *Arabidopsis lyrata* detected by Structure, with optimal number of clusters determined by the highest peak for mean ln P(D). Figure S3. Probability of assignment of individuals to their respective population for different regions.
(DOCX)

Supporting Information S2 **Microsatellite data from the ten Icelandic populations of Arabidopsis lyrata used in this study.** Data is provided in GenAlEx format, with allele sizes in base pairs.
(XLSX)
