# Supplementary material for: Contrasting Patterns of Genetic Structuring in Natural Populations of Arabidopsis lyrata Subsp. petraea across Different Regions in Northern Europe
Source: PLoS One. 2014 Sep 16;9(9):e107479. doi: 10.1371/journal.pone.0107479 (PMC4166467; doi:10.1371/journal.pone.0107479)
Supplement: Supporting Information S1 — File contains Tables S1 and S2 and Figures S1, S2, and S3. Table S1. Characteristics of microsatellite loci used to describe the genetic structure in natural populations of Arabidopsis lyrata in Iceland. Table S2. Above-ground plant and seed bank density, and estimates of genetic parameters based on 17 microsatellite markers in Icelandic, Norwegian and Swedish populations of Arabidopsis lyrata. Figure S1. Heterozygosity (H E), effective population size (Ne), allelic richness (R S) and private allelic richness (R P) in Icelandic, Norwegian and Swedish populations. Figure S2. Genetic structure of Icelandic populations of Arabidopsis lyrata detected by Structure, with optimal number of clusters determined by the highest peak for mean ln P(D). Figure S3. Probability of assignment of individuals to their respective population for different regions. (DOCX) [file pone.0107479.s001.docx]

Table S1- Characteristics of microsatellite loci used to describe the genetic structure in natural populations of *Arabidopsis lyrata* in Iceland. Four loci (*ELF3*, *ICE3*, *MSAT2*.22 and *nga112*) were not included in the genetic diversity analysis and in between habitat comparisons.

| **Multiplex 1** | Primer concentration (µM) | fluorophores | Number of alleles | Allele range |
| --- | --- | --- | --- | --- |
| *ICE6* ^2^ | 0.40 | HEX | 5 | 147-156 |
| *ICE12* ^1,2^ | 0.20 | HEX | 11 | 178-212 |
| *F20D22* ^2^ | 0.20 | HEX | 3 | 165-168 |
| *nga162* ^1,2^ | 0.20 | 6-FAM | 1 | 77 |
| *nga151* ^1,2^ | 0.20 | 6-FAM | 2 | 93-99 |
| *ICE5* ^2^ | 0.20 | 6-FAM | 2 | 166-167 |
| *AthGAPAb* ^2^ | 0.45 | NED | 4 | 113-127 |
| **Multiplex 2** |  |  |  |  |
| *AthCDPK9* ^2^ | 0.20 | HEX | 4 | 86-98 |
| *F19K23-483* ^2^ | 0.30 | HEX | 5 | 175-195 |
| *SLL2* ^2^ | 0.25 | HEX | 2 | 297-309 |
| *ICE14* ^2^ | 0.40 | 6-FAM | 8 | 214-236 |
| *AthZFPG* ^2^ | 0.50 | 6-FAM | 14 | 134-168 |
| *nga280* ^1,2^ | 0.20 | NED | 3 | 74-80 |
| *ATTS0392* ^2^ | 0.20 | NED | 4 | 136-154 |
| *ICE13* ^2^ | 0.20 | NED | 10 | 218-258 |
| **Multiplex 3** |  |  |  |  |
| *ICE11* ^2^ | 0.30 | HEX | 3 | 130-142 |
| *ELF3* ^4^ | 0.20 | HEX | 14 | 283-326 |
| *ICE7* ^2^ | 0.20 | 6-FAM | 2 | 92-95 |
| *MSAT2.22* ^3^ | 0.25 | 6-FAM | 6 | 209-224 |
| *ICE3* ^2^ | 0.10 | NED | 26 | 70-123 |
| *nga112* ^1,2^ | 0.15 | NED | 5 | 217-230 |

The primer sequences of flanking regions for microsatellite loci are presented in 1) Bell and Ecker (1994), 2) Clauss et al. (2002), 3) Loudet et al. (2002) and 4) Kuittinen et al. (2004).

Table S2- Above-ground plant and seed bank density, and estimates of genetic parameters based on 17 microsatellite markers in Icelandic, Norwegian and Swedish populations of *Arabidopsis lyrata*. *H*_E_, *F*_IS_*, R*_S_, *R*_P_*, md^2^* and *θ* represent genetic diversity, inbreeding coefficient, allelic richness, private allelic richness and mutation-scaled effective population size, respectively. None of *F*_IS_ values were significantly different from zero.

| Iceland | *H*_E_ | *F*_IS_ | *R*_S_ | *R*_P_ | *md^2^* | *θ* | Plant density (m^-2^) | Seed bank (m^-2^) |
| --- | --- | --- | --- | --- | --- | --- | --- | --- |
| Pop_2 | 0.35 | -0.01 | 2.28 | 0.14 | 23.74 | 0.41 | 0.12 | 9.80 |
| Pop_3 | 0.34 | -0.27 | 2.03 | 0.04 | 30.49 | 0.36 | 5.11 | 172.41 |
| Pop_4 | 0.39 | -0.05 | 2.48 | 0.24 | 36.53 | 0.47 | 0.19 | 5.39 |
| Pop_5 | 0.36 | -0.17 | 2.55 | 0.26 | 40.05 | 0.49 | 4.51 | 32.33 |
| Pop_15 | 0.36 | -0.07 | 2.41 | 0.1 | 32.92 | 0.60 | 0.43 | 0 |
| Pop_16 | 0.34 | -0.03 | 2.22 | 0.02 | 26.65 | 0.45 | 0.67 | 0 |
| Pop_17 | 0.34 | -0.13 | 2.11 | 0.01 | 28.48 | 0.37 | 0.23 | 10.78 |
| Pop_21 | 0.37 | -0.08 | 2.23 | 0.08 | 31.69 | 0.55 | 1.20 | 5.39 |
| Pop_22 | 0.33 | 0.05 | 2.17 | 0.13 | 39.39 | 0.53 | 0.61 | 0 |
| Pop_23 | 0.32 | 0.02 | 2.31 | 0.24 | 34.63 | 0.34 | 0.74 | 32.33 |
| Average | 0.35 | -0.08 | 2.28 | 0.13 | 32.46 | 0.46 | 1.38 | 26.84 |
| SE | 0.01 | 0.03 | 0.05 | 0.03 | 1.69 | 0.03 | 0.58 | 16.63 |
| Norway |  |  |  |  |  |  |  |  |
| N1 | 0.25 | 0.07 | 2.02 | 0.07 | 11.09 | 0.22 | 0.30 | 0 |
| N2 | 0.24 | -0.11 | 1.96 | 0.11 | 15.39 | 0.25 | 1.50 | 183.19 |
| N3 | 0.25 | -0.06 | 2.09 | 0 | 9.37 | 0.26 | 0.63 | 17.14 |
| N4 | 0.22 | 0.08 | 1.89 | 0.04 | 9.59 | 0.33 | 0.63 | 18.34 |
| N5 | 0.28 | -0.11 | 2.08 | 0.11 | 12.22 | 0.32 | 2.33 | 15.09 |
| N6 | 0.27 | -0.15 | 1.89 | 0 | 8.12 | 0.25 | 3.00 | 45.96 |
| N7 | 0.19 | -0.24 | 1.73 | 0.05 | 16.41 | 0.27 | 15.00 | 161.64 |
| N8 | 0.20 | 0.13 | 1.76 | 0 | 8.59 | 0.24 | 2.78 | 12.83 |
| N9 | 0.29 | 0.00 | 2.3 | 0.11 | 15.35 | 0.34 | 0.42 | 93.39 |
| N10 | 0.25 | 0.02 | 2.07 | 0.05 | 14.26 | 0.23 | 3.50 | 46.53 |
| N13 | 0.34 | 0.01 | 2.64 | 0.07 | 31.97 | 0.40 | 0.70 | 312.50 |
| N14 | 0.20 | -0.12 | 1.68 | 0 | 11.57 | 0.30 | 11.25 | 28.86 |
| N15 | 0.21 | 0.23 | 1.92 | 0.08 | 8.17 | 0.30 | 0.83 | 0 |
| N16 | 0.28 | 0.02 | 2.33 | 0.07 | 17.97 | 0.32 | 0.37 | 7.92 |
| Average | 0.25 | -0.02 | 2.03 | 0.05 | 13.58 | 0.29 | 3.09 | 67.39 |
| SE | 0.01 | 0.03 | 0.07 | 0.01 | 1.66 | 0.01 | 1.19 | 24.44 |
| Sweden |  |  |  |  |  |  |  |  |
| S1 | 0.33 | 0.03 | 2.21 | 0 | 19.75 | 0.30 | 0.25 |  |
| S2 | 0.36 | -0.13 | 2.32 | 0.12 | 18.17 | 0.31 | 2.00 |  |
| S3 | 0.36 | 0.00 | 2.22 | 0.01 | 15.84 | 0.35 | 2.00 |  |
| S6 | 0.27 | -0.06 | 2.05 | 0.04 | 13.39 | 0.25 | 1.20 |  |
| S7 | 0.28 | -0.09 | 1.98 | 0 | 11.26 | 0.31 | 2.67 |  |
| S8 | 0.31 | -0.06 | 2.18 | 0.06 | 25.03 | 0.40 | 0.30 |  |
| S10 | 0.35 | 0.01 | 2.41 | 0.04 | 18.55 | 0.30 | 1.25 |  |
| S13 | 0.36 | 0.10 | 2.4 | 0.01 | 17.96 | 0.32 | 2.00 |  |
| S15 | 0.38 | -0.06 | 2.42 | 0.04 | 25.71 | 0.38 | 0.38 |  |
| S16 | 0.21 | -0.25 | 1.8 | 0 | 29.34 | 0.18 | 0.20 |  |
| S17 | 0.32 | 0.05 | 2.52 | 0.18 | 22.05 | 0.44 | 1.00 |  |
| S19 | 0.38 | 0.18 | 2.28 | 0.06 | 16.03 | 0.29 | 0.67 |  |
| Average | 0.33 | -0.02 | 2.23 | 0.05 | 19.42 | 0.32 | 1.16 |  |
| SE | 0.01 | 0.03 | 0.06 | 0.02 | 1.53 | 0.02 | 0.24 |  |

Figure S1- Heterozygosity (*H*_E_), effective population size (*N*_e_), allelic richness (*R*_S_) and private allelic richness (*R*_P_) in Icelandic, Norwegian and Swedish populations. The plus sign and blue dots indicate mean value and the range of data, respectively. Dots plotted outside of whisker line are considered outliers. The lower and upper bound for each box represent 1st and 3rd quartile, respectively.

Figure S2- Genetic structure of Icelandic populations of *Arabidopsis lyrata* detected by Structure, with optimal number of clusters determined by the highest peak for mean ln P(D). a) Each pie represents membership coefficients of each population to the inferred cluster (*K* = 7). b) Membership coefficients of individuals to the inferred clusters. Solid vertical lines separate populations.
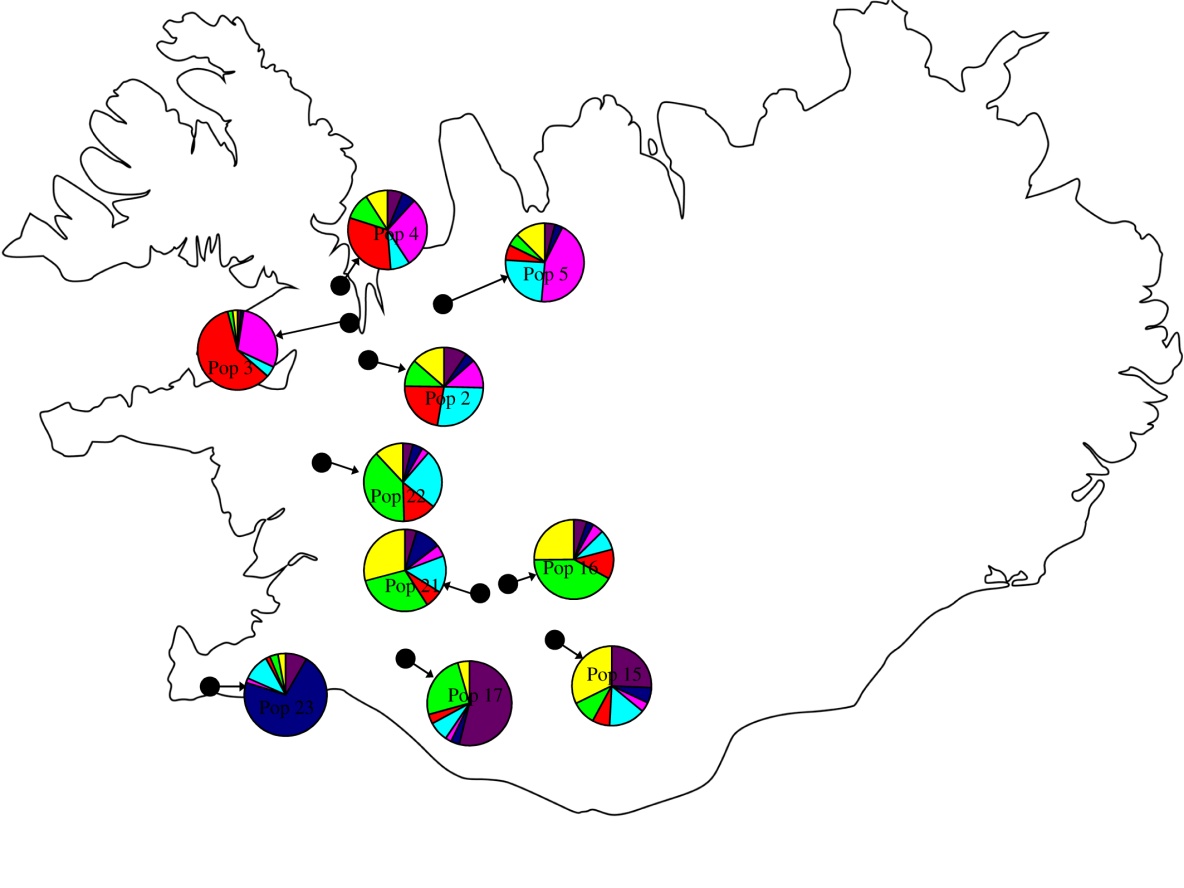


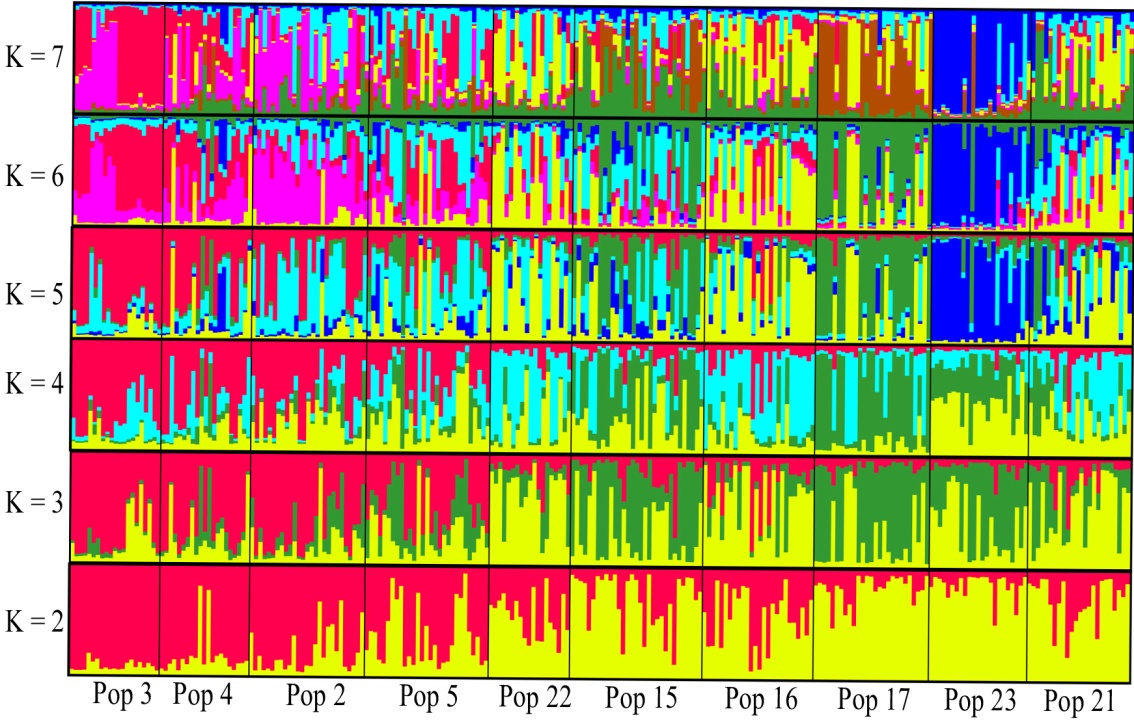


Figure S3- Probability of assignment of individuals to their respective population for different regions. The frequency of each probability class represents the number of individuals which are assigned to that class based on GeneClass2.

**LITERATURE CITED**

Bell, C. J., and J. R. Ecker. 1994. Assignment of 30 microsatellite loci to the linkage map of *Arabidopsis*. *Genomics* 19: 137-144.

Clauss, M. J., H. Cobban, and T. Mitchell-Olds. 2002. Cross-species microsatellite markers for elucidating population genetic structure in Arabidopsis and Arabis (Brassicaeae). *Molecular Ecology* 11: 591-601.

Kuittinen, H., A. A. de Haan, C. Vogl, S. Oikarinen, J. Leppälä, M. Koch, T. Mitchell-Olds, et al. 2004. Comparing the linkage maps of the close relatives *Arabidopsis lyrata* and *A. thaliana*. *Genetics* 168: 1575-1584.

Loudet, O., S. Chaillou, C. Camilleri, D. Bouchez, and F. Daniel-Vedele. 2002. Bay-0 × Shahdara recombinant inbred line population: a powerful tool for the genetic dissection of complex traits in *Arabidopsis*. *Theoretical and Applied Genetics* 104: 1173-1184.
